# Editorial: Immune Evasion Strategies in Protozoan-Host Interactions

**DOI:** 10.3389/fimmu.2020.609166

**Published:** 2020-10-30

**Authors:** Alexandre Morrot

**Affiliations:** ^1^ Tuberculosis Research Laboratory, Faculty of Medicine, Federal University of Rio de Janeiro, Rio de Janeiro, Brazil; ^2^ Immunoparasitology Laboratory, Oswaldo Cruz Foundation, FIOCRUZ, Rio de Janeiro, Brazil

**Keywords:** infection diseases, protozoan parasites, persistent infections, immune evasion, immunopathogenesis

One of the most successful protozoans is the malaria parasite, which has evolved key mechanisms to avoid the host immune system. This theme was recently reviewed with a focus on their capacity to overcome both innate responses, and the induction and maintenance of adaptive immune responses (Gomes, Bhardwaj et al.; Rénia and Goh; Gomes, Feijó et al.). With regards to this topic, the authors highlighted the importance of carbohydrate-mediated interactions that directly affect *Plasmodium* survival and host resistance. *Plasmodium* parasites have a complex life cycle in the vertebrate host. Initial stages comprise infection of hepatocytes, in which the CSP and TRAP domains of the sporozoite form of the parasite mediate an adhesive interaction with sulfated glycoconjugates on the surface of hepatocytes, initiating the intracellular parasitism stage in the host. The dependency on carbohydrate-mediated interactions for the parasitism also continues in the bloodstream form of the parasite *via* carbohydrates expressed by red blood cells, such as ABO, Lewis, and Duffy, which thereby influences erythrocyte parasitism (Gomes, Feijó et al.).

The glycobiology of parasite-host interactions has been proposed as a potential drug target. This is of particular importance for Chagas disease, a chronic infection caused by *Trypanosoma cruzi*. *T. cruzi* infection induces CD8 T cell responses able to control but not eliminate the parasite, which is then able to subvert the host defenses *via* immuno-endocrine regulation (Cardoso et al.; Morrot et al.; Mendonça et al.; Silva-Neto et al.; Decote-Ricardo et al.; Sanmarco et al.; Gil-Jaramillo et al.). Drugs designed to interfere with carbohydrate-mediated interactions during host-pathogen interplay have been shown to block the ability of *T. cruzi* to evade host immune surveillance. The parasite expresses a multifunctional enzyme called trans-sialidase responsible for catalyzing the transfer of sialic acid domains from the host glycoconjugates to mucin-like molecules on the *T. cruzi* cell surface. The sialylated domains of parasite mucins are shown to jeopardize host defenses, compromising both B and T cell-adaptive responses during infection (Nardy et al.; Freire-de-Lima et al.).

The evasion strategies employed by Trypanosomadidae involve the modulation of components that make up the large arsenal of defense mechanisms. This is seen for *Leishmania* parasites in which upregulation of L-arginine and polyamine production correlate with increased levels of immunosuppressive IL-10 and concomitant reductions in the expression of the inflammatory cytokines, IL-12, and TNF-α, in *L. donovani*-infected macrophages (Mandal et al.). The maintenance of an immunosuppressive cytokine milieu by the parasite reservoir is also demonstrated in visceral leishmaniasis in which B-1 regulatory cells contribute to sustained levels of splenic IL-10 and impairment of macrophage resistance (Arcanjo et al.). Furthermore, the manipulation of host cytokine networks by *Leishmania* parasites (Mahanta et al.) has been shown to be a determinant factor for the establishment of immune evasion mechanisms that favor parasite persistence in *Leishmania* infection (Pérez-Cabezas et al.; Falcão et al.; de Freitas et al.).

Trypanosomatids serve as good examples of parasites that modulate the contribution made by activated macrophages in controlling infection. *T. brucei*-derived trypanosome suppression immunomodulating factor (TSIF) plays a role in triggering both macrophage and T cell suppressive states thus leading to pathogenicity (Stijlemans et al.). Other host immune evasion mechanisms rely on the suppression of the macrophage respiratory burst. This is well characterized for *Entamoeba* *histolytica* parasitism as the parasite can induce neutrophil apoptosis and suppress nitric oxide (NO) production by macrophages (Begum et al.). In fact, reactive oxygen species (ROS) production seems to be a major component in the ability of macrophages to resolve protozoan parasite infections (Moreira-Souza et al.). The control of cell-mediated innate responses may impair the development of effective immune mechanisms thus allowing parasite survival (Geiger et al.). Modulation of lipid pathways is another critical step in guaranteeing protozoan parasite survival by interfering with sentinel cell signaling, clearance, and trafficking mechanisms (Toledo et al.; Totino and Lopes). This is exemplified by *Toxoplasma gondii* infection, where parasites reach immune-privileged host sites and persist in quiescence (Nakada-Tsukui and Nozaki; Chakraborty et al.; Brasil et al.).

Understanding host-protozoan interactions requires interdisciplinary study focused on these complex interactions within biological systems. In malaria, determinants of hepatocyte liver stage infection have been elucidated, and new systems biology approaches have resulted in the development of methodologies that could ultimately lead to advanced strategies to eradicate malaria (Zuck et al.). Following this approach, the characterization of the malaria parasite secretome has provided knowledge on immune evasion and virulence targets that can be used to optimize intervention strategies to control infection (Soni et al.). Finally, the outcome of protozoan-host interactions can also be influenced by co-infections (Pinna et al.), the ability of parasites to manipulate host resources and defense responses for their survival (Ramírez-Toloza et al.; Kakani et al.; Linhares-Lacerda and Morrot), and polymorphisms (Lima-Junior and Pratt-Riccio).

The eradication of protozoan infections is complex and must involve the integration of new approaches as control strategies. Successful health management policies to contain and eradicate such tropical diseases must include the integration of diverse intervention strategies (Filardy et al.). Applying geographic information systems technologies to monitor the spatial distribution of drug resistance and parasite polymorphisms (Filardy et al.), together with effective disease biomarkers for early parasite diagnosis and that distinguish clinical phases of infections (Linhares-Lacerda et al.; Pinho et al.), optimized vaccine design (Wilson et al.; Luca and Macedo; Furtado-de-Mendonça et al.; Minigo et al.; Stijlemans et al.), the use of next generation drugs and alternative therapies for progressive diseases (Pandey et al.), as well as a better understanding of the vector biology for controlling the transmission cycles (Soumana et al.; Ngoune et al.; Jacob et al.), will, together, permit us to develop effective protozoan disease elimination programs.

## Author Contributions

The author confirms being the sole contributor of this work and has approved it for publication.

## Conflict of Interest

The author declares that the research was conducted in the absence of any commercial or financial relationships that could be construed as a potential conflict of interest.

